# Customization options in consumer health information materials on type-2 diabetes mellitus—an analysis of modifiable features in different types of media

**DOI:** 10.3389/fpubh.2024.1252244

**Published:** 2024-02-21

**Authors:** Cornelia Krenn, Thomas Semlitsch, Carolin Zipp, Stefan Lengauer, Lin Shao, Tobias Schreck, Michael Bedek, Cordula Kupfer, Dietrich Albert, Bettina Kubicek, Andrea Siebenhofer, Klaus Jeitler

**Affiliations:** ^1^Institute of General Practice and Evidence-based Health Services Research, Medical University of Graz, Graz, Austria; ^2^Institute of Computer Graphics and Knowledge Visualization, Graz University of Technology, Graz, Austria; ^3^Fraunhofer Austria Center for Data Driven Design, Graz, Austria; ^4^Institute of Psychology, University of Graz, Graz, Austria; ^5^Institute of General Practice, Goethe University Frankfurt am Main, Frankfurt, Germany; ^6^Institute for Medical Informatics, Statistics and Documentation, Medical University of Graz, Graz, Austria

**Keywords:** consumer health information, type-2 diabetes mellitus, user-centered customization options, tailoring health information, health literacy

## Abstract

**Introduction:**

The understanding of health-related information is essential for making informed decisions. However, providing health information in an understandable format for everyone is challenging due to differences in consumers’ health status, disease knowledge, skills, and preferences. Tailoring health information to individual needs can improve comprehension and increase health literacy.

**Objective:**

The aim of our research was to analyze the extent to which consumers can customize consumer health information materials (CHIMs) for type-2 diabetes mellitus through various media types.

**Methods:**

We conducted a comprehensive search for various CHIMs across various media types, such as websites, apps, videos, and printed or printable forms. A representative sample of CHIMs was obtained for analysis through blocked randomization across the various media types. We conducted a quantitative content analysis to determine the frequency of user-centered customization options. Cross-comparisons were made to identify trends and variations in modifiable features among the media.

**Results:**

In our representative sample of 114 CHIMs, we identified a total of 24 modifiable features, which we grouped into five main categories: (i) language, (ii) text, (iii) audiovisual, (iv) presentation, and (v) medical content. Videos offered the most customization opportunities (95%), while 47% of websites and 26% of apps did not allow users to tailor health information. None of the printed or printable materials provided the option to customize the information. Overall, 65% of analyzed CHIMs did not allow users to tailor health information according to their needs.

**Conclusion:**

Our results show that CHIMs for type-2 diabetes mellitus could be significantly improved by providing more customization options for users. Further research is needed to investigate the effectiveness and usability of these options to enhance the development and appropriate provision of modifiable features in health information.

## 1 Introduction

Health literacy is essential for making informed health-related decisions, acting independently, and making autonomous decisions on health issues. Several factors can influence health literacy, such as education, age, gender, or country of origin. A significant determinant of health literacy is the relationship between a person’s personal abilities and the health information services available in their specific environment. Therefore, the provision of understandable health information is essential to improving health literacy (1–6). Consumer health information materials (CHIMs) are presented in diverse formats, such as written text, tabular or graphical representations, auditory information, and audiovisual formats, each providing different levels of detail of information. However, the preferred content, presentation format, and level of detail of information vary significantly between consumers (7). The challenge is to deliver health information effectively to consumers, tailored to their health status, disease knowledge, skills, and specific needs and preferences (8, 9). Despite the diversity of information needs and preferences, we hypothesize that existing CHIMs typically provide a ‘one-size-fits-all’ approach. To the best of our knowledge, current CHIMs present unfiltered information directly to the patient and do not, or only to a limited extent, allow for the selection or preselection of the preferred medical content, presentation format, and level of detail of information, aligned with individual consumer situations and needs.

However, tailored consumer health information (CHI) have the potential to increase health literacy and encourage people in appropriate self-care (9). Traditional interventions such as lectures, passive lessons, one-way delivery of information, the distribution of brochures and leaflets, and education sessions with visual aids all have the potential to increase health literacy in certain target groups (10). However, Ramsey et al. (11) suggest that CHIMs should be available in a variety of formats and not be exclusively provided via a single medium.

To date, there is a noticeable lack of research that specifically investigates the options and extent to which existing CHIMs facilitate consumers in making choices regarding medical content, presentation format, and level of detail of information. We found research that indicates the importance of tailored online health information that is customized to the consumer’s unique characteristics, needs, and preferences (12) and the importance of customized intervention in routine health care (13). However, we did not identify any study that investigated the types and extent of user-centered customization options available in different media types of CHI.

The aim of our research was to address this gap by analyzing various types of media of CHI, with a focus on those related to type-2 diabetes mellitus (T2DM), and highlighting the necessity for more implementation of user-centered customization options in CHIMs. We decided to analyze CHIMs on T2DM due to the rapidly increasing global mortality rates associated with the disease (14, 15). Furthermore, T2DM is a complex disease that tends to change over the course of the disease. The information needs of consumers need to be adapted due to the many different and long-lasting health implications (16). The findings of this analysis should help health researchers and developers of consumer health information systems to systematically conceptualize and implement user-centered customization options that enable health information to be tailored to the needs of consumers. Furthermore, the identified customization options should be used to investigate which customizations contribute to a better understanding, higher motivation to use health information, or higher usability.

## 2 Methods

### 2.1 Selection criteria

We searched for CHIMs on T2DM that explicitly target laypersons of any age, gender or social group, be they patients with T2DM, their relatives, or other persons interested in T2DM. CHIMs on T2DM were considered relevant to this research when they were provided on websites, as apps, in videos, and as printed or printable health information (p-HI) on paper or in an electronic format (PDF). We therefore included brochures, leaflets, folders, posters, patient guidelines, fact sheets, and decision aids. The search was restricted to CHIMs in German and English, and to CHIMs developed in countries the World Health Organization classifies as belonging to the low-mortality strata (17). We excluded CHIMs that targeted prediabetes, diabetes mellitus type 1, late autoimmune diabetes in adults, maturity-onset diabetes of the young, other rare types of diabetes, and women with gestational diabetes. We also excluded instructions on the use of disease-related assistive technologies and tools to facilitate disease management (e.g., cooking recipes), patient biographies, educational information for the general population, health information materials for health professionals, press releases, blogs, and books about T2DM.

### 2.2 Search strategy

The aim of the search strategy we developed was to locate a broad range of currently available CHIMs on T2DM in different types of media. The search began in April 2021 and was completed in May 2022. For websites and digital p-HI, we utilized a range of sources, including diabetes associations, diabetes organizations listed on the international diabetes federation website, national health portals, ministry websites, and reputable international health information providers. We additionally used Google to identify further websites and digital p-HI. We performed a systematic search for apps that offer CHI on T2DM in the Google Play Store (Android) and the App Store (iOS). Furthermore, we actively searched for videos on YouTube, but also included videos found on already identified websites that we added to our pool of CHIMs. To expand our pool of CHIMs, we further conducted a comprehensive search for systematic reviews in PubMed, which we then utilized as an additional source. For the various searches, keywords related to diabetes (“diabetes mellitus,” “diabetes mellitus type 2,” “diabetes type 2,” “DM type 2,” “type II diabetes,” “adult-onset diabetes”) and health information (“health information,” “patient information,” “consumer information”) were used. We also performed a local survey and contacted several diabetes outpatient clinics, primary health care units, self-help groups for T2DM, pharmacies, and members of our institute’s network for research and teaching practices to identify further CHIMs and references to them.

### 2.3 Selection of a representative sample of CHIMs

As a large amount of CHIMs were found, a representative sample was then selected from the pool of CHIMs identified during the searches. In order to generate a representative sample and to obtain a balanced selection of CHIMs, we used blocked randomization in six different groups of media: (1) websites of diabetes organizations (WDO), (2) other websites not specialized in diabetes (WOO), (3) p-HI of diabetes organizations, (4) p-HI of other organizations, (5) apps and (6) videos. The sample was supposed to be equally distributed across different types of media, and we ensured no individual publishers or media companies were overrepresented. This decision was based on the assumption that different types of media differ in the way they permit CHI to be presented. In an iterative process, we randomly selected one CHI from each of the six pre-defined groups of media simultaneously in each round of randomization (i.e., blocks of six CHIMs).

### 2.4 Data extraction

We extracted the identified modifiable features of each randomly selected CHIM. Before we started with the data extraction, we predefined features that, in our view, are possible to customize currently available CHIMs (see Table 1). With regard to sociodemographic customization options, we checked each CHIM to see if variables such as age, gender, education, and ethnicity were explicitly mentioned or considered in the content of the CHIM. For instance, the medical content can be customized by providing information on gender-specific differences related to potential adverse effects of diabetic medications as well as complications of diabetes among men and women diagnosed with T2DM. However, we adopted an open-minded approach during the data extraction process, intentionally allowing for the discovery of additional customization options that may not have been preconceived. We examined each CHIM systematically, looking out for any emerging modifiable features that may not have been explicitly predetermined. We continued data extraction until saturation was achieved, i.e., we extracted data until no new adaptable content appeared in three consecutive rounds of randomization.

**Table 1:** Predefined features for which a possible customization of CHIMs can be expected.

Modifiable feature	Example
Appropriate medical content	Relevant subfield of diabetes General or more specific information
Type of presentation	Different presentation formats, e.g., textual (e.g., running text), graphical, tabular, or interactive form
Level of detail of information	Different depth and complexity of medical content
Sociodemographic feature	Age Gender Education Ethnicity
Communicative feature	Additional language
Technical feature	Display elements like Font style Font size Font color
Situational feature	Current health status Current therapeutic situation
Other feature	Not predefined

### 2.5 Method of analysis

Our analysis method followed a quantitative content analysis, commonly used to identify patterns or trends in large data sets. The analysis encompassed the following steps:

1. Categorization of user-centered customization options: to establish a structured foundation for analysis, we systematically categorized the identified modifiable features from our representative sample. This categorization enabled us to group similar features into main customization categories, providing a comprehensive understanding of different modifiable features.
2. Quantitative measurement of frequencies: the second task involved a systematic count of the occurrences of each feature, providing numerical insights into the prevalence of customization options across various types of media. A Microsoft Excel worksheet was created to record the frequency of modifiable features extracted from each medium.
3. Cross-comparison of frequencies across different media types: we conducted cross-comparisons to capture differences in modifiable features among the six types of media. This aimed to recognize trends and variations in user-centered customization options and understand differences in the presentation of CHIMs. The frequencies were also analyzed in Excel. The main objective of our analysis was to determine the number of main categories of customizations, along with their possible modifications, and the number of CHIMs that offer at least one customization.

## 3 Results

**Figure 1:**
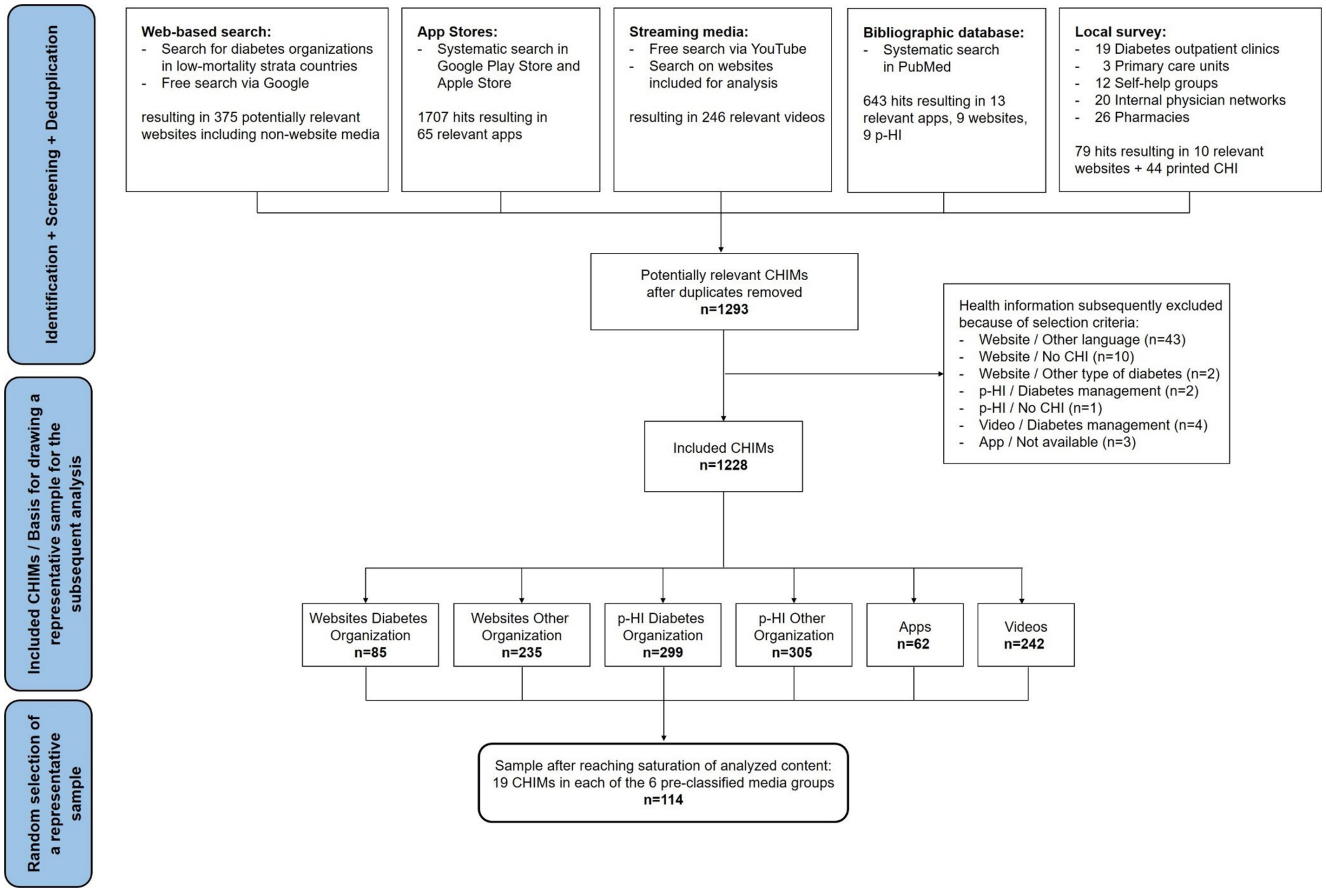
Flowchart of search for CHIMs and selection of the representative sample on T2DM.

Overall, our search identified 1,293 CHIMs related to T2DM. After removing duplicates, we found 1,228 relevant CHIMs. The response rate from our local survey was 4%. The majority of CHIMs on T2DM were p-HI from organizations that were not specialized in diabetes (*n* = 305), while apps (*n* = 62) provided the fewest relevant CHIMs. See Figure 1 for a flowchart illustrating the detailed search process. We achieved data saturation after a total of 19 randomization rounds, i.e., 19 CHIMs were extracted from each of the pre-classified six media groups. Thus, our representative sample consisted of 114 CHIMs, which we used for the subsequent analyses.

### 3.1 Overview of user-centered customization options in different types of media

In our representative sample, we identified different possibilities for users to customize health information presented on websites, in apps, and in videos. We could not determine any possibilities for users to customize p-HI. Overall, we identified a total of 24 modifiable features, which we grouped into five main categories (see Table 2). The number of modifiable features varied across the different main categories and ranged from 1 to 17.

**Table 2:** Main categories of customization options and modifiable features in CHIMs.

Main customization categories	Modifiable features
Language customization	Language of content Language of subtitles
Text customization	**Font and background:** Font (style, size, color, opacity) Text background (color, opacity) Text field (color, opacity) Text spacing Character edge style Screen masking (highlighting the reading lines and shading the rest of the text) Subtitles (show/hide)
	**Text-to-speech:** Read aloud only Read-aloud text highlighted Automatic scrolling of read-aloud text Reading speed Reading volume
Audiovisual customization	Playback speed Audio transcription
Presentation customization	Type of presentation (same information presented in different ways, e.g., textual, tabular, graphical or audiovisual)
Medical content customization	Level of detail of content Prioritization of content

### 3.2 Overall frequency of user-centered customization options in different types of media

In our representative sample, almost two-thirds (65%) of the included CHIMs did not offer any customization options. Table 3 presents a detailed overview of the frequency of user-centered customization options identified across the different types of media.

**Table 3:** User-centered customization options in different types of media.

		Types of media of CHI					
		WDO (*n* = 19)	WOO (*n* = 19)	p-HI DO (*n* = 19)	p-HI OO (*n* = 19)	App (*n* = 19)	Video (*n* = 19)^a^
**Main categories of customizations and modifiable features**	**Language customization**	**6 (32%)**	**8 (42%)**	**0**	**0**	**4 (21%)**	**15 (79%)**^**b**^
	Language of content	6 (32%)	8 (42%)	0	0	4 (21%)	0
	Language of subtitles	na	na	na	na	0	15 (79%)^b^
	**Text customization**	**2 (11%)**	**4 (21%)**	**na**	**na**	**2 (11%)**	**15 (79%)**^**b**^
	***Font and background***	2 (11%)	3 (16%)	na	na	1 (5%)	15 (79%)
	Font style	0	2 (11%)	na	na	0	15 (79%)^b^
	Font size	2 (11%)	3 (16%)	na	na	1 (5%)	15 (79%)^b^
	Text spacing	0	2 (11%)	na	na	0	0
	Font color	0	2 (11%)	na	na	1 (5%)	15 (79%)^b^
	Font opacity	0	0	na	na	0	15 (79%)^b^
	Character edge style	0	0	na	na	0	15 (79%)^b^
	Color of the text background	0	2 (11%)	na	na	1 (5%)	15 (79%)^b^
	Opacity of the text background	0	0	na	na	0	15 (79%)^b^
	Screen masking	0	2 (11%)	na	na	0	0
	Color of the text field	0	0	na	na	0	15 (79%)^b^
	Opacity of the text field	0	0	na	na	0	15 (79%)^b^
	Show or hide subtitles	na	na	na	na	0	15 (79%)^b^
	***Text to speech***	1 (5%)	3 (16%)	na	na	1 (5%)	na
	Read aloud only	1 (5%)	3 (16%)	na	na	1 (5%)	na
	Highlighted read-aloud text	1 (5%)	2 (11%)	na	na	0	na
	Automatic scrolling of reading text	1 (5%)	2 (11%)	na	na	0	na
	Reading speed	1 (5%)	2 (11%)	na	na	0	na
	Reading volume	1 (5%)	2 (11%)	na	na	0	na
	**Presentation customization**	**0**	**2 (11%)**	**0**	**0**	**0**	**na**
	**Audiovisual customization**	**na**	**na**	**na**	**na**	**1 (5%)**	**18 (95%)**
	Playback speed	na	na	na	na	0	18 (95%)
	Audio transcription	na	na	na	na	1 (5%)	15 (79%)
	**Medical content customization**	**1 (5%)**	**2 (11%)**	**0**	**0**	**0**	**na**
	Level of detail of information	0	1 (5%)	0	0	0	na
	Prioritization of information	1 (5%)	1 (5%)	na	na	0	na
**Total customization options across all main categories, *n* (%** ^**c**^ **)**		**9 (12%)**	**16 (21%)**	**0**	**0**	**7 (7%)**	**48 (84%)**
**Total modifiable features, *n* (%** ^**d**^ **)**		**14 (4%)**	**38 (10%)**	**0**	**0**	**9 (2%)**	**198 (65%)**

a YouTube videos (*n* = 18).

b Change of subtitle.

c Calculation refers to all possible adaptable categories.

d Calculation refers to all possible modifiable features.

CHI: consumer health information, DO: diabetes organization, na: not applicable, OO: other organization, p-HI: printed/printable health information, WDO: website of diabetes organization, WOO: website of other organization.

We found that videos most often (79–95%) provided user-customization options across all of their modifiable categories. These primarily entailed adding subtitles to YouTube videos (18 out of 19; 95%) and adjustable settings relating to language and font/background. In addition, the user could change the playback speed of the video and display automatically generated audio transcriptions with timestamps.

We identified modifiable features in a total of 5 out of 19 (26%) apps. Apps provide primarily the possibility to customize the language (21%) and text (11%) of the health information. In one app (5%), audio transcriptions of videos within the app were made available to users.

We found opportunities for some form of user customization on a total of 18 of 38 (47%) websites. The majority of modifications were related to language (32–42%) and text (11–21%). Customizations regarding the medical content were only available on some websites (5–11%). Regarding the modifiable features, a non-diabetes website of a national health portal provided the highest number (12 out of 20; 60%), which included options to change the language and text, such as font style, font size, text spacing, font, text background color, the chance to highlight reading lines, and the conversion of written text into units of speech.

Across all types of media in our representative sample, only 15% of all possible modifiable features were actually available as user-centered customization options, with videos having the highest implementation rate (65%), followed by websites (7%). Apps had the lowest implementation rate (2%) and p-HI provided no modifiable features.

#### 3.2.1 Number of possible customizations across main customization categories in different types of media

Our assessment revealed that it is theoretically not possible to implement the identified user-centered customization options from the main categories in all media types. Regarding the number of categories in which customization options were available, we observed that apps were the only medium in which at least one change could theoretically be carried out in all five main categories (see Table 4). Although digital information materials are more adaptable than print materials, the incorporation of customization options in p-HI would be possible in some categories, e.g., by providing content in two different languages in a two-column layout or providing more detailed and specific health information in a separate info-box.

**Table 4:** Possible customization options in the main categories in different types of media.

	Possible customization according to media type			
Main customization categories	Website	p-HI	App	Video
Language customization	✓	✓	✓	✓
Text customization	✓	x	✓	✓
Audiovisual customization	x	x	✓	✓
Presentation customization	✓	✓	✓	x
Medical content customization	✓	✓	✓	x
**Number of main categories with possibilities for customization**	**4**	**3**	**5**	**3**

**Figure 2:**
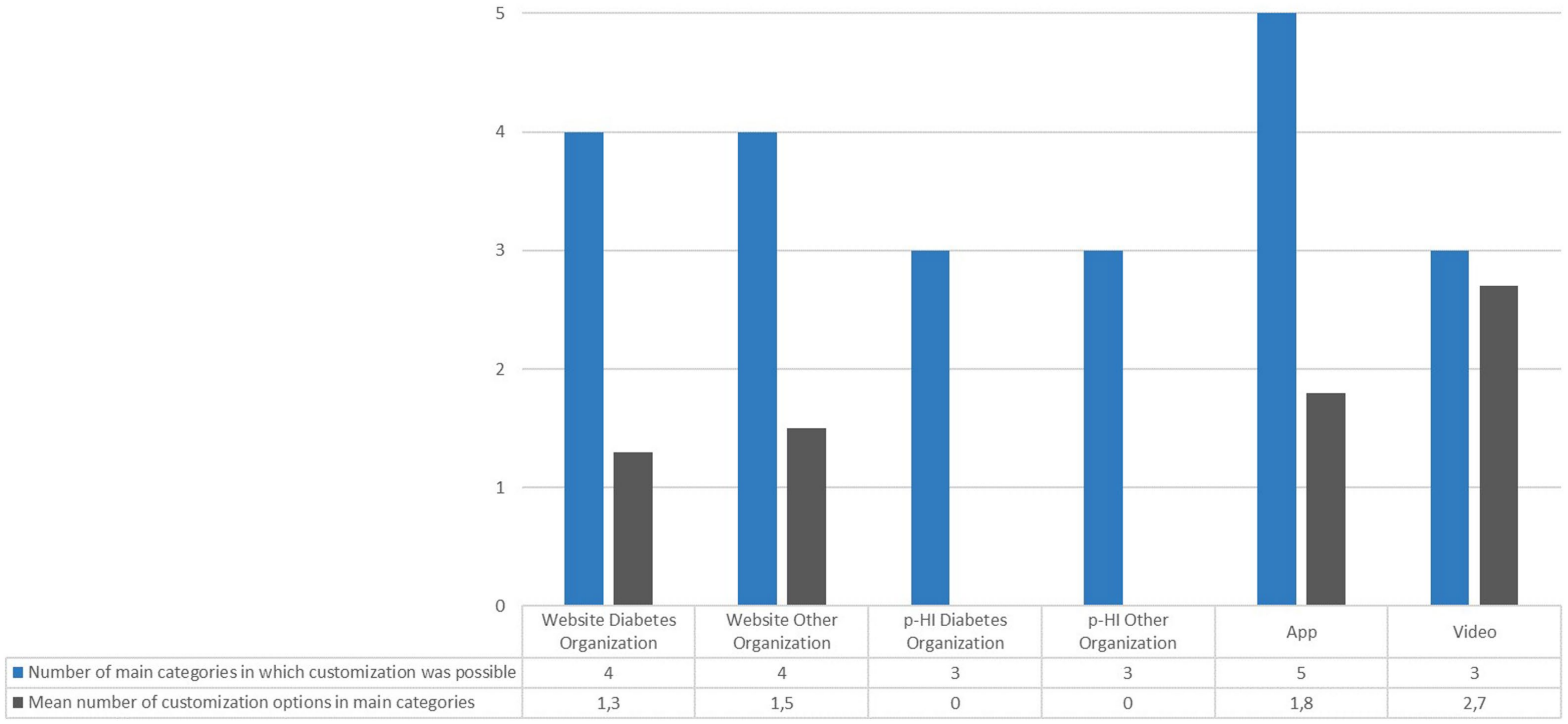
Mean number of customization options in the main categories in which it was theoretically possible.

Overall, with the exception of videos, the mean number of modifiable features in the categories where it was theoretically possible was low. In terms of the average number of categories for which websites actually provided customization options, we found no obvious difference between the websites of diabetes organizations and websites that did not specialize in diabetes (Figure 2). While most videos (15 out of 19; 79%) offered customization options in all possible categories, the websites or apps only did so in a maximum of two categories and therefore did not exploit the full range of possibilities.

#### 3.2.2 Number of at least one customization option across the different types of media

**Figure 3:**
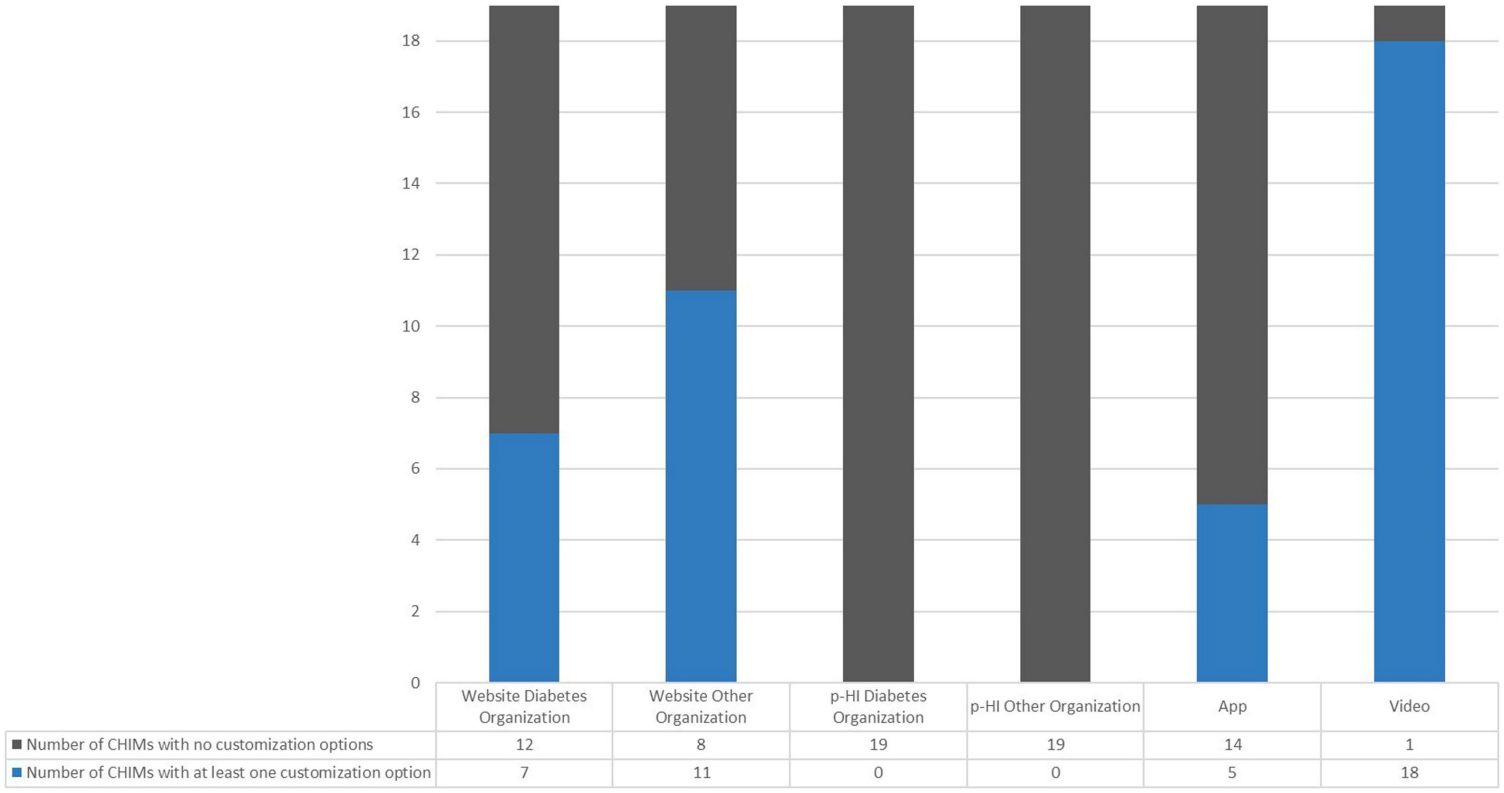
Number of CHIMs without versus at least one customization option in different media.

In our representative sample, we determined a disparity in the implementation of at least one customization option across the different media types. Figure 3 illustrates the number of CHIMs that offered at least one customization option versus those that did not. In particular, only 5 out of the 19 analyzed apps (26%), provided users with the opportunity to make any adjustments, whereas almost all of the videos (18 out of 19; 95%) did. Additionally, we identified a discrepancy in the occurrence of customization options among websites, i.e., 11 out of 19 WOOs (58%) provided some form of modification, while only 7 out of 19 WDOs (37%) did so.

### 3.3 Characteristics and limitations of user-centered customization options in CHIMs

The identified customization options were mainly found in videos and in the categories of language, text, and audio-visualization. We could not identify an option to adjust CHIMs for sociodemographic and situational features.

#### 3.3.1 Language customization

On some of the websites and apps in our representative sample, users could choose between 2 and 20 different languages, with websites generally providing more opportunities to switch between different languages. However, the majority of the websites (WOO: 58%; WDO: 68%) and apps (79%) did not translate the whole or even selected content into different languages.

The selection of language was made possible through language buttons, drop-down menus, or text links. Symbols such as a national flag, globe, location icon, or letter symbols indicated a possible language switch. However, a specific icon or symbol to indicate the option to change the language was not generally provided on websites or in apps. Some CHIMs (7 out of 18; 39%) did not offer language switching options in the target language, e.g., Deutsch, Français, and 日本語 rather than German, French, or Japanese. On one website, it was not possible to switch back to the original language after it had been changed.

Moreover, most websites and apps did not indicate how they translated their health information, i.e., whether they had employed a professional human translation service or a machine translation tool (e.g., DeepL or Google Translate). In contrast to websites and apps, users accessing health information on YouTube could make use of an automatic translation service for subtitles and captions in more than 100 different languages.

#### 3.3.2 Text customization

We identified several text-based modification options for barrier-free accessibility for users. However, in our representative set of CHIMs, these were rarely used. The customization options included changes of font style, font size, font and background color combinations (e.g., yellow text on a black background), as well as spacing up the text, and screen masking. Various options were used to change these display elements. Users were able to adjust the font size of either the whole page or a specific section of a page. Two websites had integrated a web-based support tool allowing users to change the font style, font size, text spacing, color of the font and background, and screen masking. The YouTube platform had the highest percentage of text-based modification options (79%), providing the opportunity for users to customize video subtitles by adjusting the font style, font size, font color, font opacity, text spacing, character edge style, as well as the color and opacity of the text background and text field.

In our representative sample, a total of five CHIMs (four websites, one app) had integrated a text-to-speech reader. A text-to-speech reader is an assistive technology that converts digital text into units of speech and then reads them aloud to the user (18). This technology is helpful for blind or visually impaired users, for people with dyslexia and other cognitive, learning, or reading disabilities, and for those who prefer to listen (19). Some readers have varying degrees of functionality, such as the option to automatically convert the written text into a synthesized computer voice, or even into lifelike speech that matches the patterns and tone of a natural-sounding voice. Furthermore, the page automatically scrolled up and down, and highlighted the corresponding text while the audio was playing, providing users with a complete overview of the webpage. Users could also have the text read aloud at different reading speeds and volumes, as required.

#### 3.3.3 Audiovisual customization

Audiovisual customization options included adjusting playback speeds and accessing audio transcriptions for both audio and video content. In our sample, all the videos provided by YouTube were available at up to eight different playback speeds, and audio transcriptions could be provided with timestamps. Audio transcriptions were also available in one app for videos of highlighted diabetes topics.

#### 3.3.4 Presentation customization

We found only 2 out of 38 websites (5%) that presented data in a number of different ways. One of these websites featured an interactive diabetes surveillance system that allowed users to modify visualizations of geographic trends in diabetes and its risk factors at national, state, and county levels, based on age, sex, ethnicity, and education. The system included maps, tables, graphs, and motion charts, enabling users to view the diabetes data in various ways simultaneously or separately. The second website presented data on the prevalence and incidence of diagnosed diabetes in the form of running text, bar charts, time series, and tables. On this website, the different presentation formats are presented simultaneously, and users see all formats in parallel.

#### 3.3.5 Medical content customization

The possibility to choose the preferred level of detail of medical content, as well as to filter and prioritize medical content, was only rarely provided. On one website, users were able to choose between two different levels of detail of information, ranging from a summary to more detailed information on T2DM screening. Two websites included a filtering function based on an algorithmic prioritization mechanism aimed at providing individualized CHI. The algorithm matched the user’s individual interests and information needs after generating a profile based on questions about the individual diabetes situation.

## 4 Discussion

The results of our research confirmed the hypothesis that existing CHIMs on T2DM predominantly employ one-size-fits-all user interfaces and provide few options for users to select or pre-select the preferred medical content, the form of presentation, and the level of detail of information according to their individual situation and the needs of the consumer.

In our representative sample of 114 CHIMs, we identified a total of 24 different modifiable features. We grouped these features into five main customization categories: language, text, audiovisual, presentation, and medical content. In our view, it would be possible for existing CHIMs on T2DM to provide a wide range of customization options. However, we observed that the implementation of the identified possibilities was limited. We did not find a single CHIM across various types of media (websites, apps, p-HI, and videos) that provided customization options in all the categories in which it would have been theoretically possible. We found that videos provided the most opportunities, while none of the p-HI in our sample offered any customization options. However, the identified customization options in videos did not affect the content of the information but rather the visualization of the subtitle. Our recommendation for future consumer health information systems is to present videos in a linear fashion, including menu navigation.

We further observed that the mean number of theoretically possible options in the main categories was low on websites and in apps. Our results show that CHIMs tend to offer simpler features, such as language and text customization more frequently than more complex ones, such as the opportunity to customize presentation formats and medical content. This may be because the implementation of complex adaptations requires more advanced technological solutions, while the implementation of simpler adjustments is less challenging. Furthermore, the implementation may be less challenging as long as the content requires no modification.

We found that several CHIMs in our representative sample offered health information in a single language. However, language barriers can impede individuals’ understanding of health-related information. Research has shown that people with an immigrant background or low literacy skills often have lower levels of health literacy and poorer access to healthcare services. As a result, they are more likely to engage in unhealthy behaviors, be less aware of their health, and use the health care system more intensively (20). Moreover, people with an immigrant background may feel that they are not provided with sufficient health information or treated differently than the general population (21). A recent study found that immigrants prefer multilingual health information, and that their preferred sources of health information are general practitioners (75%) and the internet (58%) (20). It is therefore essential to develop digital, user-friendly, and multilingual CHIMs in plain language and easy-to-understand formats for people with low health literacy (20–23). Furthermore, patients with an immigrant background have indicated that culture-sensitive adaptations (e.g., cover pictures, social themes, specific metaphors/idioms, or names of people in the respective country) to CHI are more useful than standard translated CHI. This effect was greater in patients with low dominant society immersion (24). In our representative sample, some websites used machine translation tools to translate their CHI into multiple languages. Although these tools partially fulfill the need to communicate health information multilingually, their accuracy remains limited, with accuracy declining in line with sentence complexity. It is advisable to pre-edit medical terminology and use lay terms in order to increase the accuracy of translations. Additionally, translations from English into western European languages are of higher quality than those into such languages as Chinese. Many machine translation initiatives are still in a pilot stage and should be used for translations of health information with caution (25–28). Some websites in our CHIM-set used country flags as an icon to highlight language switching options. However, flag icons should be avoided for language navigation, as flags represent countries rather than languages, and many countries share a language, or have more than one official language. An alternative to flag icons might be the use of translated language names and language names in the native language [e.g., German version (Deutsch) or the use of ISO language codes, such as ES, FR, DE, etc.] (29–32). Furthermore, multilingual websites and apps should always provide the option for users to switch back to the original language (33).

People with visual impairments or other disabilities may encounter further barriers when browsing the web, such as visual (e.g., color blindness), acoustic (e.g., hearing loss), and cognitive barriers. To address these barriers, the Web Content Accessibility Guideline (WCAG) 2.1 (19) provides a wide range of recommendations to help website developers improve the accessibility of web content for individuals with disabilities that include text-based and multimedia customization options. Our results show that only a small percentage of the websites and apps we analyzed followed the recommended criteria, such as the option to modify font and background settings or provide text-to-speech tools. However, while our analysis was not primarily focused on investigating the accessibility of web content, recent research has found that websites providing CHI do not have adequate levels of accessibility (34–37).

In our analysis, we observed few opportunities to adjust the presentation of the same information. Research has provided evidence that decision aids are effective in improving an individual’s functional health literacy, which is essential for shared decision-making (38–42). However, differences in individual preferences, capabilities, knowledge, attitudes, and behaviors mean the optimal way to present CHI (e.g., textual, tabular, graphical, or audiovisual) varies between consumers (41, 43–45). Informed decision-making therefore necessitates the provision of different formats and alternative ways of presenting CHI (46). Furthermore, graphics, such as pictograms and diagrams can be used to complement the presentation of numerical data in text or tables (23). According to a systematic review, pictorial health information improves knowledge retention and the recall of health information among patients of all literacy levels (47). Buljan et al. (48) found that consumers considered infographics to be more user-friendly and reported a more satisfactory reading experience than text-based lay summaries of health information. Consumers also reported being less satisfied with written health information than students and doctors. However, these effects were not statistically significant (48). The optimal type of presentation format of health information can depend on the audience, and individual preferences can differ between consumers (48).

The use of the internet for seeking CHI has rapidly proliferated in recent years (49). In addition, the internet became even more important as a source of information during the Covid-19 pandemic (50). However, some patient groups generally prefer printed CHIMs to digital ones. The preference for a specific medium is influenced by various factors, including, age, education level and familiarity with technology. Older patients with low health and computer literacy were more likely to prefer printed materials. Digital CHI sources are becoming more popular but patients with a preference for digital CHI tend to be younger and more tech-savvy (51–54). The majority of consumer health information systems will be provided digital in the future, and the focus of research about user-centered customization options should be on this media type. However, printed CHIMs should also be made available with a greater degree of customizations.

A recent systematic review found that video animations are also an enjoyable CHI-tool. Moe-Byrne et al. (55) demonstrated the positive effects of video animations on knowledge in comparison to easy-to-read information, standard printed information, real-time or static images, and audio-recorded information.

We identified only two websites that offered the possibility of prioritizing health information according to the consumer’s needs and preferences. To enhance patient knowledge and empowerment, research has highlighted the need to prioritize health information to take into account individual characteristics and preferences (56–60). The use of a personalized and modifiable diabetes information portal has been shown to improve patient satisfaction and to promote a focus on essential information. Based on the patient’s profile, irrelevant health information (e.g., information about insulin for patients who do not need it) can be removed, and health information can be prioritized based on factors that have the most significant impact on the disease outcome, the patient’s anticipated knowledge of the disease, and the patient’s information preferences. Results also indicate that the prioritization of CHI depends on the stage of the disease, with recently diagnosed patients preferring less tailored information, while patients in an advanced stage may prefer more detailed information due to their prior knowledge. Patients said that the level of detail of health information could be provided via links from less exhaustive information to external websites (60, 61). As oversimplified plain language summaries of medical research articles do not always guarantee a better reading experience, they may be regarded negatively by some consumers. In contrast, written summaries using medium-complexity wording have the potential to increase reader engagement (43).

To the best of our knowledge, this is the first research to analyze customization options in existing CHIMs on T2DM in different types of media. However, some limitations to our analysis must be acknowledged. First, as we only analyzed 114 CHIMs, our representative sample was relatively small. However, we used *a priori* defined criteria to end data analysis, and based on these criteria, we reached saturation. Furthermore, it was restricted to CHIMs on T2DM, and we excluded CHIMs on other medical topics or diseases. Our search for CHIMs was further limited by language restrictions, focusing only on CHI in German and English. Additionally, the search for CHIMs was also restricted to countries with low mortality rates. These factors may limit the generalizability of our findings. Furthermore, our search for relevant videos was restricted to YouTube and did not consider alternative streaming media platforms. Finally, we did not investigate the accessibility of web content on websites and in apps based on the WCAG 2.1 recommended success criteria (19).

## 5 Conclusion

Our results indicate that the ability of CHIMs to meet individual needs and preferences could be significantly improved by expanding the range and accessibility of customization options as far as the media format under consideration permits it. Future studies should investigate the effectiveness of different customization options and their impact on user accessibility, engagement, and health literacy, as well as the potential barriers for implementing more complex customization options.

## Author contributions

CKr: conceptualization, methodology, formal analysis, investigation, writing – original draft, and preparation. ThS: conceptualization. CZ: investigation. SL, LS, MB, and CKu: writing – review & editing. ToS and DA: funding acquisition and writing – review & editing. BK: writing – review & editing and supervision. AS: funding acquisition and supervision. KJ: conceptualization, methodology, formal analysis, writing – review & editing, and supervision. All authors contributed to the article and approved the submitted version.
